# Protocol for a prospective, cluster randomized trial to evaluate routine and deferred dialysis initiation (RADDI) in Chinese population

**DOI:** 10.1186/s12882-019-1627-0

**Published:** 2019-12-09

**Authors:** Xinju Zhao, Pei Wang, Lining Wang, Xiaonong Chen, Wen Huang, Yonghui Mao, Rihong Hu, Xiaohong Cheng, Caili Wang, Li Wang, Ping Zhang, Detian Li, Yuzhu Wang, Wenling Ye, Yuqing Chen, Qiang Jia, Xiaoyan Yan, Li Zuo

**Affiliations:** 1 Department of Nephrology, Peking University People’s Hospital, Beijing, China; 2 Department of Nephrology, The First Affiliated Hospital of Zhengzhou University, Zhengzhou, China; 3 Department of Nephrology, The First Affiliated Hospital of China Medical University, Shenyang, China; 4 Department of Nephrology, Ruijin Hospital Affiliated to Shanghai Jiao Tong University, Shanghai, China; 5 Department of Nephrology, Beijing Tongren Hospital Capital Medical University, Beijing, China; 6 Department of Nephrology, Beijing Hospital, National Center of Gerontology, Beijing, China; 7 Department of Nephrology, Hangzhou Hospital of Traditional Chinese Medicine, Hangzhou, China; 8 Department of Nephrology, Shaanxi Hospital of Traditional Chinese Medicine, Shaanxi, China; 9 Department of Nephrology, The First Affiliated Hospital of Baotou Medical College, Baotou, China; 10 Department of Nephrology, Sichuan Academy of Medical Sciences, Chengdu, China; 11 Kidney disease center, The First Affiliated Hospital, College of Medicine, Zhejiang University, Hangzhou, China; 12 Department of Nephrology, Shengjing Hospital of China Medical University, Shenyang, China; 13 Department of Nephrology, Beijing Haidian Hospital (Beijing Haidian Section of Peking University Third Hospital), Beijing, China; 14 Department of Nephrology, Peking Union Medical College Hospital, Beijing, China; 15 Renal Division, Peking University First Hospital, Beijing, China; 16 Department of Nephrology, Xuanwu Hospital Capital Medical University, Beijing, China; 17 Peking University Clinical Research Institute, Beijing, China

## Abstract

**Background:**

The timing of when to initiate dialysis for progressive chronic kidney disease (CKD) patients has not been well established. There has been a strong trend for early dialysis initiation for these patients over the past decades. However, the perceived survival advantage of early dialysis has been questioned by a series of recent observational studies. The only randomized controlled trial (RCT) research on this issue found the all-cause mortality, comorbidities, and quality of life showed no difference between early and late dialysis starters. To better understand optimal timing for dialysis initiation, our research will evaluate the efficacy and safety of deferred dialysis initiation in a large Chinese population.

**Methods:**

The trial adopts a multicenter, cluster randomized, single-blind (outcomes assessor), and endpoint-driven design. Eligible participants are 18–80 years old, in stable CKD stages 4–5 (eGFR > 7 ml/min /1.73 m^2^), and with good heart function (NYHA grade I or II). Participants will be randomized into a routine or deferred dialysis group. The reference eGFR at initiating dialysis for asymptomatic patients is 7 ml/min /1.73 m^2^ (routine dialysis group) and 5 ml/min/1.73 m^2^ or less (deferred dialysis group) in each group. The primary endpoint will be the difference of all-cause mortality and acute nonfatal cerebro-cardiovascular events between the two groups. The secondary outcomes include hospitalization rate and other safety indices. The primary and secondary outcomes will be analyzed by appropriate statistical methods.

**Discussion:**

This study protocol represents a large, cluster randomized study evaluating deferred and routine dialysis intervention for an advanced CKD population. The reference eGFR to initiate dialysis for both treatment groups is targeted at less than 7 ml/min/1.73m^2^. With this design, we aim to eliminate lead-time and survivor bias and avoid selection bias and confounding factors. We acknowledge that the study has limitations. Even so, given the low-targeted eGFR values of both arms, this study still has potential economic, health, and scientific implications. This research is unique in that such a low targeted eGFR value has never been studied in a clinical trial.

**Trial registration:**

The trial has been approved by ClinicalTrials.gov (Trial registration ID NCT02423655). The date of registration was April 22, 2015.

## Background

Over the past two decades, the number of uremic patients that has received renal replacement therapy (RRT) has increased worldwide [1]. According to a rough estimate, the number of dialysis patients in China in 1999 was 42,000 cases and increased rapidly to 608,000 cases in 2015 [2]. The annual cost of dialysis treatment alone reached 429 million RMB in 2015 [2]. The rapid increase of dialysis patients has brought a heavy economic burden on Chinese society.

The strong trend of early dialysis initiation for end-stage kidney disease (ESKD) patients over recent decades makes the burden even greater. Data from the United States showed that the proportion of new dialysis patients with glomerular filtration rate (GFR) ≥10 ml/min/1.73m^2^ increased rapidly from about 10% in 1996 to around 50% in 2008 [3]. Between years 2000–2004 and 2005–2009, mean estimated GFR (eGFR) at dialysis initiation increased from 9.8 to 11.0 ml/min/1.73 m^2^ [4]. Early dialysis initiation not only increases the cost of medical treatment but also has no additional benefit for patients in long-term outcomes. Observational data showed that the short-term or long-term survival was not affected for chronic kidney disease (CKD) patients with stable low GFR (≤5 ml/min/1.73m^2^, and even ≤2 ml/min/1.73m^2^) before dialysis treatment [5, 6]. A recent retrospective cohort study found no statistically significant survival difference for early compared with later dialysis initiation (interpolated eGFR at dialysis therapy initiation ≥10 ml/min/1.73m^2^ vs. < 10 ml/min/1.73m^2^) [7]. Furthermore, some data even showed that early initiation seemed to produce an additional burden to patients and the health care system [5, 6, 8]. The Initiating Dialysis Early and Late (IDEAL) study, the only RCT research on this issue, found that the all-cause mortality, comorbidities, and quality of life showed no difference between early (GFR 10–14 ml/min/1.73m^2^) and late (GFR 5–7 ml/min/1.73m^2^) dialysis starters [9]. However, this study is limited by the condition that the difference for average GFR between two groups (12 ml/min/1.73 m^2^ vs. 9.8 ml/min/1.73m^2^) was not great. Moreover, the achieved GFR were relatively high for both groups. Thus, several major renal organizations have reassessed the risks and benefits of the practice of initiating early RRT [10–14]. The Canadian Society of Nephrology released a clinical practice guideline in which an “intent-to-defer” approach for dialysis initiation and strategy to initiate dialysis in the absence of symptoms in patients with an eGFR of 6 ml/min /1.73 m^2^ or less was recommended [12]. In this guideline, the specialists also expressed that the optimal management of patients with an estimated GFR of 6 ml/min per 1.73 m^2^ or less was based on limited data. The updated KDOQI (Kidney Disease Outcomes Quality Initiative) guideline emphasized that the decision to initiate maintenance dialysis “should be based primarily upon an assessment of signs and/or symptoms … rather than on a specific level of kidney function in the absence of such signs and symptoms.” [14]

The timing for initiating dialysis for ESKD patients is an important issue yet has not been well established, therefore, we have designed the study “Routine and Deferred Dialysis Initiation (RADDI)” to evaluate the efficacy and safety of deferred dialysis initiation.

## Methods

### Study design

**Fig. 1:**
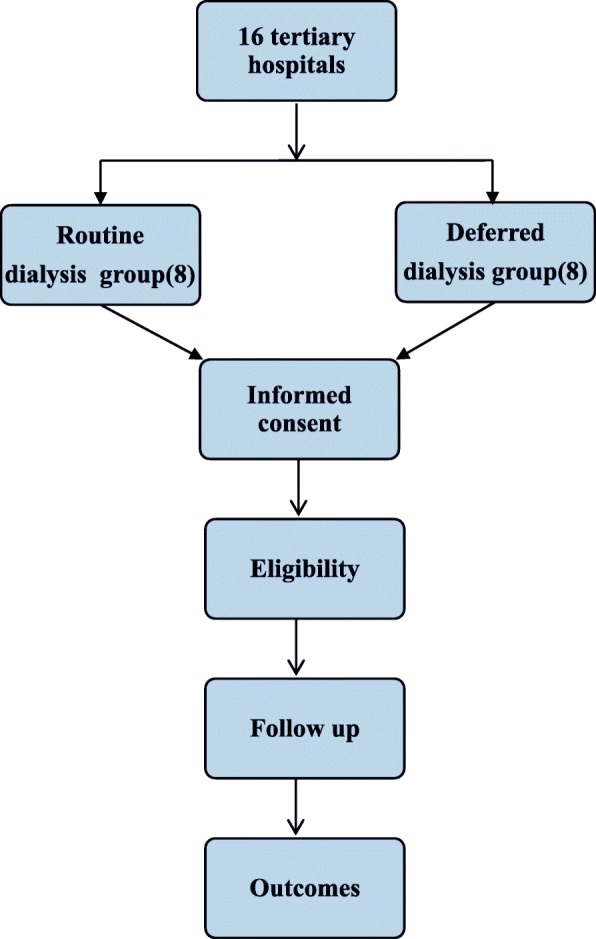
Diagram of the study design

The RADDI trial (the newest protocol version was 4.0, OUPM2017-12-25 on 25 Dec 2017) is a parallel assignment, prospective, single-blind (outcomes assessor), multicenter trial involving 16 clinical centers in China (see Fig. 1. Diagram of the study design).

### Main inclusion criteria

1. Participants aged 18–80 years old
2. Nondialysis-dependent stable CKD stage 4–5 patients (eGFR > 7 ml/min /1.73 m^2^)
3. Heart function: grade I or II (NYHA Functional Classification)

### Main exclusion criteria

Subjects who meet one of the following conditions shall not be included in the trial:

1. Patients with estimated short life expectancy (due to causes other than kidney disease);
2. Acute infection occurred within the past month;
3. Myocardial infarction, stroke events, or being diagnosed with heart function of NYHA class IV within the past 3 months;
4. Uncontrolled malignancy;
5. Active viral hepatitis;
6. Active rheumatic disease;
7. Pregnant women, women intending to conceive after enrollment or breastfeeding women;
8. Patients scheduled to have transplantation during the study period;
9. With indices requiring emergency dialysis;
10. eGFR less than 7 ml/min/1.73m^2^ in first visit;
11. Participating in other clinical studies that could impact this study;
12. Unable to provide written informed consent.

### Trial intervention and visit schedule

#### Randomization

Cluster randomization was utilized. The intervention was applied at the facility level to avoid the possibility of experimental contamination at the patient level. Sixteen tertiary hospitals across China have been randomly assigned to the routine and deferred dialysis groups using a random number generator. At each site, on referral to the study, participants will be sequentially allocated a unique identifying number to be used for all subsequent study documentation. This will ensure confidentiality is maintained.

#### Blinding

Assessors for the primary outcome in the study will be blinded for the duration of the trial. Assessors for the secondary outcomes will not be blinded.

#### Pre-inclusion visit

Eligible patients will be asked to give their informed consent. The CKD-EPI (Chronic Kidney Disease Epidemiology Collaboration) formula is used to estimate GFR for participants (based on serum creatinine, as shown in Table 1). The pre-inclusion visit allows each putative inclusion to be validated via selection and validation procedures by the responsible researchers. Once approved by the selection and validation procedures, patients will undergo the inclusion visit.

**Table 1:** CKD-EPI formula used to estimate GFR

Gender	serum creatinine (Scr)	CKD-EPI formula
female	≤0.7 mg/dL	eGFR = 144 × (Scr/0.7)^-0.329^X(0.993)^age^
	Scr > 0.7 mg/dL	eGFR = 144 × (Scr/0.7)^-1.209^X(0.993)^age^
male	Scr ≤0.9 mg/dL	eGFR = 141 × (Scr /0.9)^-0.411^X(0.993)^age^
	Scr > 0.9 mg/dL	eGFR = 141 × (Scr /0.9)^-1.209^X(0.993)^age^

### Inclusion visit

During the inclusion visit, baseline laboratory tests will be obtained for patients eligible for this trial.

### Intervention

#### Routine dialysis initiation group

**Fig. 2:**
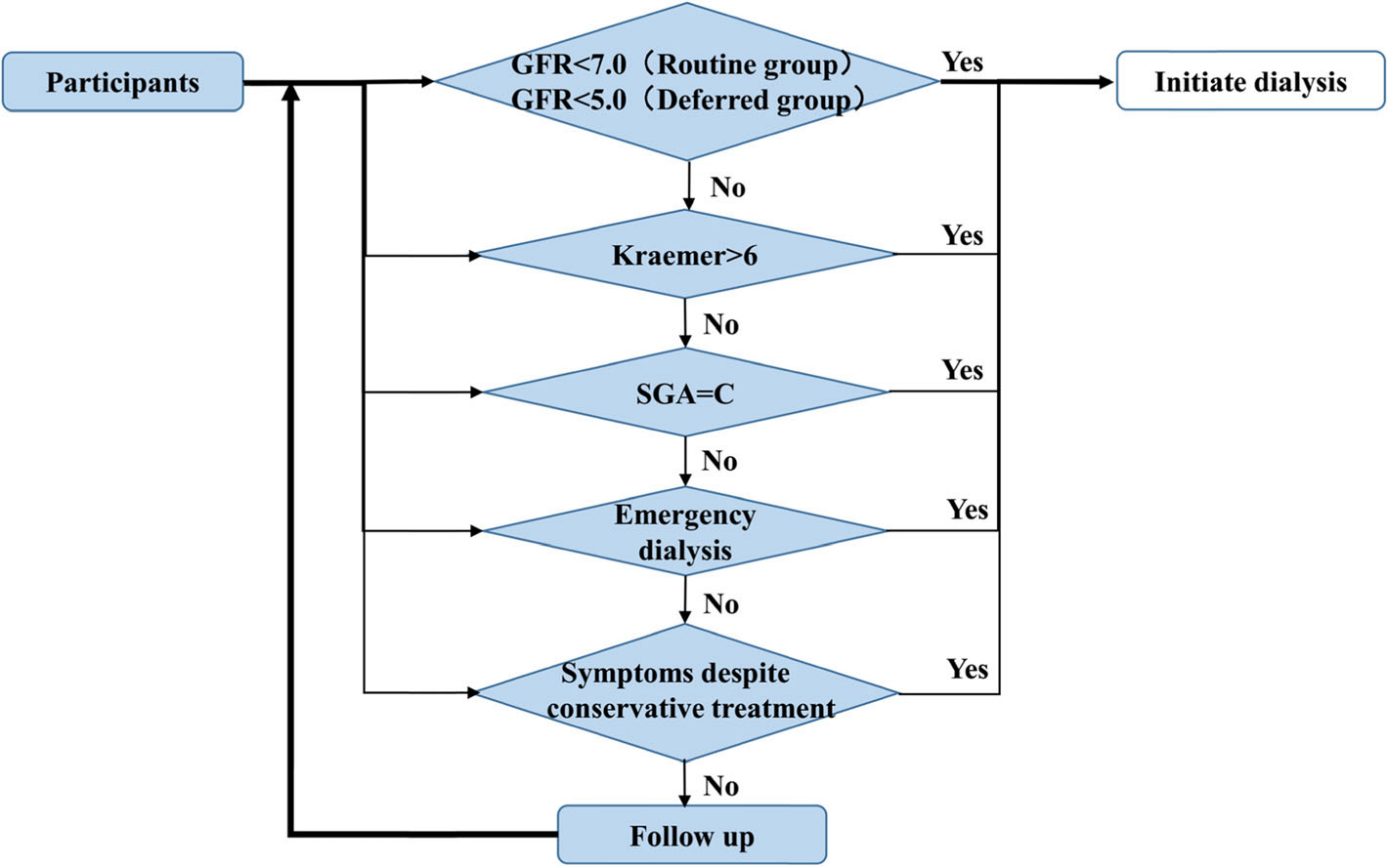
The flow paradigm of clinical indicators for dialysis initiation

In this control group, the decision whether or not to initiate dialysis in progressive CKD patients will be made based on a flow paradigm of clinical indicators (Fig. 2). Researchers will start dialysis treatment when a patient’s eGFR reaches 7 ml/min/1.73m^2^ in asymptomatic patients. Alternatively, researchers will initiate dialysis when patients have clinical indications listed below:

1. Patient’s modified Kraemer index is > 6 [15] (Table 2) or with overt fluid overload after trying all conservative means (including appropriate medicines);
2. Patients with subjective global assessment (SGA) assessment grade C;
3. Patients with indications for emergency dialysis (Table 3);
4. Patients with severe symptoms, which cannot be relieved by conservative treatment.

**Table 2:** The modified Kraemer index to evaluate symptoms of hypervolemia

Clinical score of symptoms of hypervolemia	
Systolic Blood pressure ≥ 180 (new onset)	+ 2
Pretibial edema, weak	+ 2
Chronic coughing (new)	+ 2
Dyspnoea at rest, recumbent	+ 2
Pretibial edema, severe	+ 3
Dyspnoea at rest, one cushion	+ 3
Dyspnoea at rest, two cushions	+ 4
Dyspnoea at rest, sitting	+ 6

**Table 3:** Indications for emergency dialysis

Patients meet any of the following conditions may begin emergency dialysis after adequate drug therapy:	
(1)oliguria or anuria for more than 2 days	
(2)hyperkalemia: blood potassium > 6.5 mmol/L	
(3)carbon dioxide combining power (CO_2_CP) < 13 mmol /L	
(4)acute pulmonary edema	
(5)Gallop rhythm of the heart	

#### Deferred Dialysis initiation group

In this intervention group, similar flow paradigm of clinical indicators (Fig. 2) for initiating dialysis are used. Researchers will not start dialysis treatment for patients who are asymptomatic until their eGFR is below 5 ml/min/1.73m^2^. If safe for patients, investigators will postpone dialysis as late as GFR 2 ml/min/1.7m^2^. Clinical indications for initiating dialysis are the same as listed above for the routine dialysis initiation group.

### Follow-up

All patients will attend a study visit every 3 months before and after dialysis is performed.

For each visit, a clinical and biological evaluation will be performed (Table 4). Special tests such as body composition (not mandatory, only for sites having the device), ultrasound cardiography (UCG), chest X-ray, kidney ultrasound will be taken annually. The well-validated short form health survey questionnaire 36(SF-36) survey instrument will assess health-related quality of life. Nutritional status of patients is evaluated using the SGA scale, which includes medical history and a physical examination. Medical history consists of 5 categories: (1) history of weight loss, (2) dietary intake change, (3) gastrointestinal symptoms, (4) functional capacity, and (5) metabolic demand related to the underlying disease. Physical examination focuses in the detection of muscle wasting, loss of subcutaneous fat, and the presence of edema. Based on this information, according to the subjective evaluation of the observer, the nutritional diagnosis is defined and the patients are classified as: (A) well-nourished, (B) moderately (or suspected of being) malnourished, or (C) severely malnourished. Serious adverse events, acute nonfatal cerebro-cardiovascular events, and hyperkalemia will be reported prospectively. Moreover, the cost for patient spending on CKD disease will be recorded.

**Table 4:** Schedule for visits

Parameters recorded	Pre-inclusion visit	inclusion visit	Before dialysis		Initiation dialysis	After dialysis	
			Quarterly visit	every 6 months visit		Quarterly visit	every 6 months visit
Medical history	X						
Symptoms	X	X	X		X	X	
Physical evaluation	X	X	X		X	X	
SGA		X		X	X		X
QOL		X		X	X		X
CBC		X	X		X	X	
Alb		X	X		X	X	
Scr	X	X	X		X	X	
eGFR	X	X	X		X	X	
SI and Ferritin		X		X	X	X	
iPTH		X		X	X	X	
BNP		X		X	X		X
CRP		X		X	X		X
DW					X	X	
UF					X	X	
UO					X	X	
Clinical events			X		X	X	
Costs			X		X	X	

*SGA* subjective global assessment, *QOL* quality of life (by short form 36), *CBC* complete blood count, *Alb* Albumin, *Scr* serum creatinine, *eGFR* estimated glomerular filtration rate, *SI* serum iron, *iPTH* Intact parathyroid hormone, *BNP* brain natriuretic peptide, *CRP* C reactive protein, *DW* dry weight, *UF* ultrafiltration amount, *UO* urine output. Physical evaluation includes vital signs (blood pressure, heart rate, body weight, height, etc.), degree of edema and other positive signs

### Endpoints

#### Primary endpoint

The primary endpoint will be all-cause mortality and acute nonfatal cerebro-cardiovascular events before and after dialysis initiation. We will compare proportions of all-cause death rates and acute nonfatal cerebro-cardiovascular events before and after dialysis treatment between two groups.

#### Secondary endpoints

The secondary outcomes will allow the investigators to:

- Assess the hospitalization rates by comparing proportion of patients who are admitted to hospital before and after dialysis treatment between the two groups;
- Assess the nutritional status before and after dialysis treatment between the two groups by using SGA assessment and serum albumin levels;
- Assess patient reported quality of life by using SF-36.
- Assess the complications of dialysis after dialysis treatment between the two groups;
- Implement a cost study.•Assess arteriovenous fistulas rate and catheter usage as blood access at first dialysis.

### Economic evaluation

#### Cost measurement

To assess the total cost of each group, the number of resources consumed will be prospectively collected for each patient (drugs, medical devices, consultations, hospitalization, etc.). The receipts from each visit will be used to record the patient’s costs during the follow-up period.

### Statistical analysis

#### Sample size

In this study, a cluster randomized design is used. The primary endpoint of the study is death rate, which will be determined by survival analysis. According to the IDEAL study, the death rate was 37% during a median follow-up period of 3.59 years [9]. Because we include relatively stable patients the mortality rate may be lower; we assume the death rate will be 30% or less within 3 years. The project selection window for patient enrollment is 30 months. Sood et al. found an adjusted facility-level intraclass correlation coefficient (ICC) of 0.031 in their work [16]. We get 8 clusters per group. Scholars have proposed that a sample size calculated assuming individual randomization can be inflated by a Design Effect (DE) to reach the required level of statistical power under cluster randomization [17]: DE = 1+ (m-1) ρ, where m is the number of individuals per cluster and ρ is the ICC.

If we assume that alpha = 5%, (1-beta) = 80%, patients are assigned 1:1, a difference of 10% in death rates between these two groups is considered acceptable. We use log-rank tests to get the events (87 vs 57) with the Power Analysis and Sample Size Software, version 13 (NCSS, LLC. Kaysville, Utah, USA) and the number of individuals per group is 473. Thus, the number of total events under the consideration of cluster effects is 198 (128 vs 70). This is an endpoint driven study. We will complete the study when we get enough endpoint events, which is 198 events.

#### Analysis

In this randomized controlled trial, equivalence tests for two proportions in a cluster-randomized design will be performed according to requirements in the CONSORT (Consolidated Standards of Reporting Trials) statement. Sociodemographic, clinical, and economic data will be analyzed per group.

Baseline characteristics at enrollment will be reported as the mean and standard deviation (SD) for continuous variables and percentage for categorical variables. Continuous variables not normally distributed will also be presented as a median and interquartile range.

Missing data will be imputed using multiple imputation regression methods.

Cox analysis will be used to compare all-cause mortality, acute nonfatal cerebro-cardiovascular events, and hospitalization before and after dialysis treatment between the two groups.

T-tests will be performed to compare nutritional status, quality of life, residual renal function (urine volume), and medical expenses based on a data-paired analysis (before-after study). Continuous data will be compared using a paired t-test if the variable is normally distributed or Wilcoxon test for non-parametric variables. The MacNemar test will be used for categorical variables (Fleiss test, if necessary).

Statistical significance will be considered at a value of *p* ≤ 0.05. All statistical analyses will be performed with SAS, version 9.4 (SAS Institute, Cary, NC; USA).

### Study management

#### Selection committee

A selection committee composed of investigators from each center will review the medical history and the indications for inclusion in the RADDI protocol.

#### Monitoring

A contract research organization (CRO) has been hired for this study. The coordinator will ensure that the study is conducted in accordance with the International Council for Harmonization of Technical Requirements for Pharmaceuticals for Human Use (ICH) standards of good clinical practice (GCP) through site monitoring visits. The CRO provides an online website to enter data that includes range checks for data values. The website can also act as a reminder for the investigator to follow up with patients. The monitoring plan has been written and agreed to. The data-monitoring committee has been established to monitor 100% of the data.

### Safety

#### Adverse events

Any adverse events (AE) occurring during the course of this study must be recorded in the corresponding case report form (CRF). In addition, the participants will be asked questions about the occurrence of AE during the researchers’ examination of vital signs and blood collection. “Clinical adverse events” refers to a disease, signs or symptoms that occur during the course of the study. All the clinical AE, whether or not they are considered to be relevant to the study, will be documented and described on the AE page of the CRF by the investigator. The names of the disease or disorder will be recorded (i.e. diagnosis). The researchers will provide the participants with the results of the laboratory tests that are considered clinically significant and will be recorded on the AE page of CRF. All clinically significant abnormal laboratory results, whether related to this study, will be reported. Abnormal laboratory results that are considered serious will be reported promptly and the patient will be treated appropriately. For all adverse events, the researchers are required to follow up with the patient until the events are eliminated or stabilized and to record the outcomes of the events.

## Discussion

Optimal timing for chronic dialysis initiation in ESKD patients is currently unknown. The transition period from the pre–ESKD phase to the ESKD phase of CKD is critical for patients. In our previous study, we found that mortality rates in the first 2 months were highest for new chronic dialysis starters (41.9 and 16.6 per 100 patient-years) [18]. Determining optimal timing for dialysis initiation is of great importance. This study protocol represents a large, randomized study evaluating the efficacy and safety of deferred and routine dialysis intervention for an advanced CKD Chinese population. The IDEAL study demonstrated no significant difference in survival between treatment groups. However, the average GFR were 12.0 ml/min/1.73m^2^ in the early start group and 9.8 ml/min/1.73m^2^ in the late start group, which were relatively higher than those recommended in recently released guidelines. In China, because of limited medical resources, dialysis is routinely started for ESKD patients at a much lower eGFR. In our study, the reference eGFR for initiating dialysis is 7 ml/min/1.73m^2^ or lower. No interventional study has targeted such a low reference eGFR to date. This study will improve our understanding of the timing of chronic dialysis.

Our study is a cluster randomized trial. As pointed out, designing interventions targeting delayed dialysis initiation at the facility level might be difficult in the United States, because dialysis facilities do not usually participate in the decision to initiate dialysis [4]. In China, nephrologists in large hospitals make the decision to dialyze ESKD patients and then provide treatment in their affiliated dialysis facilities. Thus, this enables us to design our research to be carried out at the facility level. Since we follow CKD patients from earlier CKD stages, the lead-time and survivor bias can also be eliminated. Randomized trials are the gold standard method to measure the effects on outcomes [19]. Thus, this design can enable us to overcome inherent shortcomings in observational studies such as selection bias and confounding factors.

Recently, the Canadian Kidney Knowledge Translation and Generation Network published its study protocol “Knowledge Translation Interventions to Improve the Timing of Dialysis Initiation: Protocol for a Cluster Randomized Trial,” which aimed to evaluate the efficacy and safety of a knowledge translation intervention to promote the intent-to-defer approach in clinical practice [20]. The study designs have some similar components. However, they are very different. The emphasis of the Canadian research is to determine whether active knowledge translation intervention can reduce early dialysis starts. However, our study aims at evaluating the efficacy and safety of early and late dialysis initiation and the influence of timing on subsequent clinical outcomes directly.

An endpoint event-based power estimation is adopted in this study. It will allow all sites to finish together and avoid trial power being affected by alterations in accrual times. It will also protect the study power from lower than predicted event rates.

We acknowledge the following study limitations: (1) The results of the randomized study have limited generalizability due to the healthier nature of patients who participate in trials; (2) There may be a tendency for patients enrolled in clinical trials to alter their behavior due to awareness of being observed [19]. Thus, all patients may have relatively better outcomes; (3) Although this is facility-level research aimed at avoiding experimental contamination, the facilities in the routine group may also adopt the strategies in the delayed group unintentionally; (4) Patients in the routine group may actively delay their dialysis due to poor financial conditions. Thus, the eGFR rates may be similar at the end of the study and may endanger the power of this study; and (5) Patients with financial problems (e.g. without medical insurance) in both groups may get suboptimal medical care, which may impact their outcomes.

Even if the eGFR values of patients in both groups at dialysis initiation are low and not sufficiently different, this study will still potentially have economic, health, and scientific implications. Completing this study will add new knowledge about the timing of dialysis initiation.

## Data Availability

Not applicable.

## Abbreviations

AE: Adverse events
Alb: Albumin
AV: Arteriovenous fistulas
BNP: Brain natriuretic peptide
CBC: Complete blood count
CKD: Chronic kidney disease
CKD-EPI: Chronic kidney disease epidemiology collaboration
CONSORT: Consolidated standards of reporting trials
CRF: Case report form
CRO: Contract research organization
CRP: C reactive protein
DE: Design effect
DW: Dry weight
eGFR: Estimated GFR
ESKD: End stage kidney disease
GFR: Glomerular filtration rate
ICC: Intraclass correlation coefficient
IDEAL: The Initiating Dialysis Early and Late study
iPTH: Intact parathyroid hormone
KDOQI: Kidney disease outcomes quality initiative
NYHA: New York Heart Association
QOL: Quality of life
RADDI: Routine and deferred dialysis initiation
RCT: Randomized controlled trial
RRT: Renal replacement therapy
Scr: Serum creatinine
SD: Standard deviation
SGA: Subjective global assessment
SI: Serum iron
UCG: Ultrasound cardiography
UF: Ultrafiltration amount
UO: Urine output
